# Externally imposed electric field enhances plant root tip regeneration

**DOI:** 10.1002/reg2.59

**Published:** 2016-08-20

**Authors:** Nicolas Kral, Alexandra Hanna Ougolnikova, Giovanni Sena

**Affiliations:** ^1^ Department of Life SciencesImperial College LondonLondonUK

**Keywords:** Plant regeneration, plant root, Arabidopsis

## Abstract

In plants, shoot and root regeneration can be induced in the distinctive conditions of tissue culture (*in vitro*) but is also observed in intact individuals (*in planta*) recovering from tissue damage. Roots, for example, can regenerate their fully excised meristems *in planta*, even in mutants with impaired apical stem cell niches. Unfortunately, to date a comprehensive understanding of regeneration in plants is still missing. Here, we provide evidence that an imposed electric field can perturb apical root regeneration in *Arabidopsis*. Crucially, we explored both spatial and temporal competences of the stump to respond to electrical stimulation, by varying respectively the position of the cut and the time interval between excision and stimulation. Our data indicate that a brief pulse of an electric field parallel to the root is sufficient to increase by up to two‐fold the probability of its regeneration, and to perturb the local distribution of the hormone auxin, as well as cell division regulation. Remarkably, the orientation of the root towards the anode or the cathode is shown to play a role.

## Introduction

A broad biological and physical understanding of the regeneration of damaged tissues and whole organs in plants will dramatically advance our ability to control morphogenesis in multi‐cellular systems.

Plant fragments cultured *in vitro* can be induced to regenerate new shoot or root tissue by adding specific ratios of the phytohormones auxin and cytokinin to the medium (Skoog & Miller 1957). A complex transcriptional dynamics and epigenetic regulation underlies such *in vitro* regeneration, drawing important parallels with other instances of plant organogenesis (Sugimoto et al. 2010; He et al. 2012; Liu et al. 2014; Kareem et al. 2015).

Roots can regenerate their stem cell niches (Xu et al. 2006) or their whole apices *in planta* without the requirement of any exogenous phytohormone (Feldman 1976; Francis 1978; Rost & Jones 1988; Sena et al. 2009). When grown on the surface of solid medium plates, *Arabidopsis* root apices regenerate with a probability that quickly decreases with the distance of the excision point from the apex (Rost & Jones 1988; Sena et al. 2009). This defines a spatial gradient of regeneration competence, whose establishment and maintenance remains poorly understood to date. Remarkably, the regeneration of fully excised root apices occurs even in *Arabidopsis* mutants with impaired apical stem cell niches (Sena et al. 2009).

Although inhibition of either cell division or the phytohormone auxin's polar transport has been shown to abort root apex regeneration in *Arabidopsis* (Sena et al. 2009), the exact molecular mechanism underlying root regenerationcompetence remains unknown.

To address this, we asked what kind of other molecular or physical perturbations affect root regeneration *in planta*.

It has been shown that long exposure to weak electric current, both constant (Rathore & Goldsworthy 1985) and alternating (Cogalniceanu et al. 1998), can increase the efficiency of *in vitro* regeneration in tobacco tissue cultured in shoot‐inducing medium. Although there is a long tradition of exploring the effects of electric stimulation on the regeneration of animal tissues and organs (Smith 1974; McCaig et al. 2005; Levin 2007; Chang & Minc 2014), little attention has been focused on similar perturbations applied to organ regeneration *in planta*.

In the present study, we quantified the effects of an external electric field on the regeneration of root tips *in planta*. We used the primary root of *Arabidopsis thaliana* as the model system, and calculated the probability of regeneration of its fully excised apex, in the presence or absence of an imposed constant electric field.

## Results

### Root regeneration in the absence of electric field (mock conditions)

**Figure 1:**
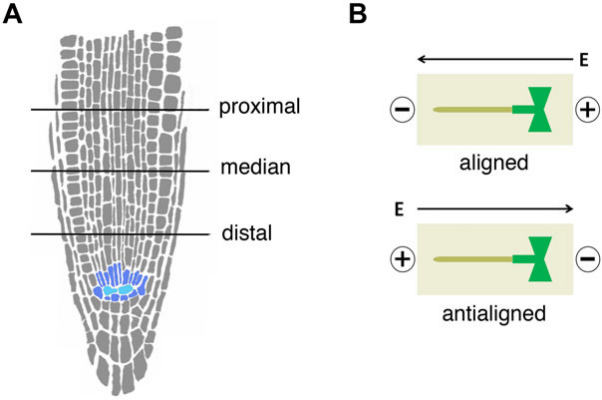
Excision points and root orientations. (A) Cartoon of *Arabidopsis* root tip showing the location of the stem cell niche (quiescent centre, light blue; initial cells, dark blue) and the position of the excision points. Distance from the root's tip: proximal, 200 μm ± 10 μm; median, 160 μm ± 10 μm; distal, 120 μm ± 10 μm. (B) Orientations of root and electric field (*E*): aligned, root tip pointing towards the negative electrode; anti‐aligned, root tip pointing towards the positive electrode.

We developed a novel setup to test the effects of externally imposed electric fields on *Arabidopsis* root tip regeneration. In each experiment, a large number of 3‐day post‐germination wild‐type plantlets had their root manually excised at an arbitrary distance from the tip to remove the apical region of the meristem (Fig. 1A), following established methods (Sena et al. 2009). After a set interval of time (RT, resting time) a sub‐set of these plantlets were then immersed in a modified electrophoresis tank filled with liquid medium and exposed for 30 min to an aligned or anti‐aligned electric field (Figs 1B, S1), while the rest of the plantlets remained immersed for the same amount of time in a second identical tank but without electric field (mock). Both tanks were equipped with active circulation of the liquid medium to maintain a uniform distribution of ions and constant temperature (Fig. S1). All plantlets were then transferred back to standard solid medium plates and monitored for root meristem regeneration until 5 days after the excision. The full removal of the apical tissue columella results in loss of gravitropism (growth alignment with gravity vector) (Barlow 1974), so that regeneration can be scored by looking at roots with re‐established gravitropism and confirmed by morphology (Sena et al. 2009). In each experiment, the probability – or the frequency – of regeneration was estimated from the sample by calculating the proportion between the number of regenerated roots and the total number of excised roots; the error of the estimate was calculated with the standard error of the proportion (Glantz 2005).

Our setup for imposing electrical stimulation requires the roots to be immersed in liquid medium for 30 min, which may result in mild hypoxic conditions. We first asked if the spatial gradient of regeneration competence observed on solid medium (Sena et al. 2009) was still detectable in our setup and in the absence of the electric field (mock). The estimated regeneration frequency for roots cut in the distal region of the meristem (*d* = 120 μm ± 10 μm) was significantly higher (chi‐squared test, *P* < 0.005 Bonferroni‐corrected) than that for roots cut in the median region (*d* = 160 μm ± 10 μm) (Fig. S2), which was also significantly higher (chi‐squared test, *P* < 0.005 Bonferroni‐corrected) than the estimated frequency in the proximal region(*d* = 200 μm ± 10 μm) (Fig. S2). Interestingly, this was true irrespective of the resting time RT between cut and submersion in the liquid medium, as confirmed by the chi‐squared statistical test not detecting any significant difference between RT = 30 min, 80 min, and 160 min when cuts were performed in the distal, median, or proximal region (Fig. S2).

These results indicate that the brief immersion in liquid medium alone in our assay does not disrupt the gradient of regeneration competence, regardless of the resting time adopted.

### A strong electric field inhibits root tip regeneration

The literature on the effects of external electric fields on plant roots is sparse. Notably, damage to maize root meristems was observed after 3 h of exposure to a constant 5.0 V/cm (Ishikawa & Evans 1990), 3.0 V/cm (Stenz & Weisenseel 1991) or 1.5 V/cm (Wawrecki & Zagorska‐Marek 2007) field but not with 0.5 V/cm (Stenz & Weisenseel 1991). In our first set of experiments, given the fact that we were going to use a brief 30‐min exposure, we applied a field of 5.0 V/cm (measured current, between 200 and 220 mA) to root tips cut in the median meristematic region (*d* = 160 μm ± 10 μm) after RT = 80 min. In these conditions the regeneration frequency *f*_E_ of the stimulated roots drastically dropped compared to the mock *f*_mock_, both for aligned and anti‐aligned orientations (Fig. S3). This result indicates that a 30‐min exposure to a 5.0 V/cm field is sufficient to inhibit regeneration competence of the root meristem.

### A weak electric field enhances root tip regeneration

We decreased the electric field strength to 2.5 V/cm (measured current, between 100 and 130 mA) and explored the spatial competence of the tissue to respond to the electric field by cutting in the distal, median, or proximal region respectively of the root meristem, at a distance *d* of 120 μm ± 10 μm, 160 μm ± 10 μm, and 200 μm ± 10 μm from the root apex (Fig. 1A). Note that all these cuts resulted in the complete removal of the root apical stem cell niche, as previously described (Sena et al. 2009).

Although the process of regeneration is believed to start at the instant the tissue is damaged, it is not known when the regenerating stump is more likely to be affected by an external electric field. To specifically address this point, we explored the temporal competence of the stump to respond to the stimulation by varying the amount of time (RT) between excision and exposure in our experiments. We tested early, mid, and late regeneration steps, respectively, at RT = 30, 80, and 160 min. To maintain high temporal resolution in our assay, and therefore to probe accurately the three regeneration steps, we adopted 30‐min stimulations in all experiments.

The electric field was applied parallel to the root longitudinal axis, and both orientations were tested: “aligned” with the root tip pointing towards the negative electrode, and “anti‐aligned” with the tip towards the positive electrode (Fig. 1B).

**Figure 2:**
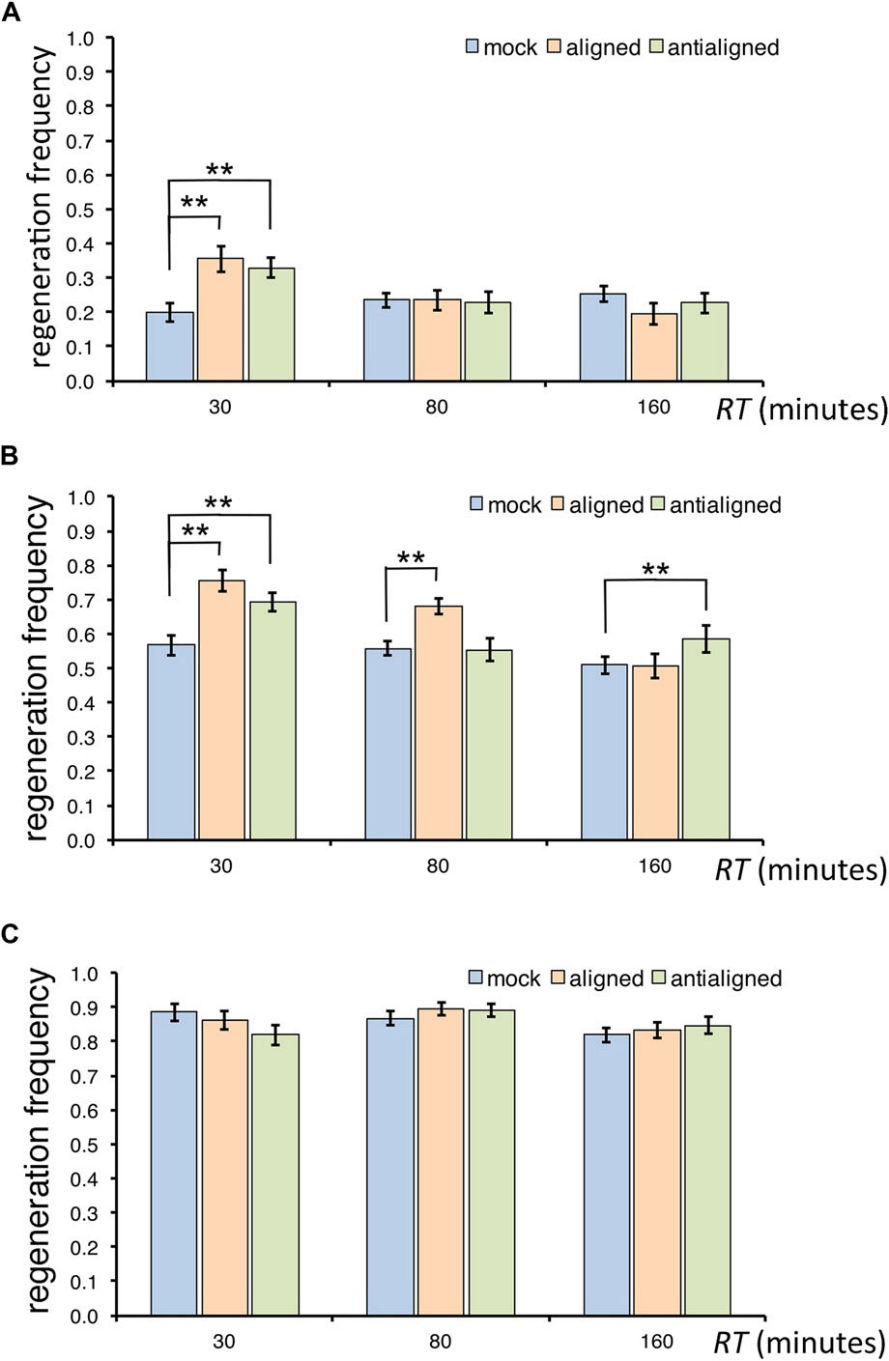
Effects of electric field on the frequency of root tip regeneration. Number of regenerated roots divided by the total number (reported in Table S1) of root tips cut at the proximal (A), median (B), and distal (C) positions and exposed to 2.5 V/cm, for resting times RT = 30, 80, and 160 min. Mock, aligned, and anti‐aligned configurations are presented color‐coded. Chi‐squared test used to compare proportions; **high significance with *P* < 0.005 Bonferroni‐corrected; error bars, standard error of the proportion.

We systematically explored the nine combinations for spatial and temporal competence: *d* × RT = {120 μm ± 10 μm, 160 μm ± 10 μm, 200 μm ± 10 μm} × {30 min, 80 min, 160 min} (Fig. 2). After calculating the regeneration frequency in each condition, we quantified the effect of the electric field by defining a regeneration ratio (RR), equivalent to the common relative risk (Glantz 2005), as the ratio of the two estimated regeneration frequencies *f*_E_/*f*_mock_ and calculated its 95% confidence interval CI_RR_ (Glantz 2005).

The most dramatic effect was found in the proximal region of the meristem (*d* = 200 μm ± 10 μm) when exposed 30 min after the excision (Fig. 2A, RT = 30 min), where regeneration frequency was estimated to increase by 80% (RR = 1.80, CI_RR_ = [1.31, 2.53]) in the aligned configuration and by 65% (RR = 1.65, CI_RR_ = [1.23, 2.32]) in the anti‐aligned configuration. Interestingly, roots cut at the same position *d* but exposed later in the process (RT > 30 min) regenerated with the same probability as those not exposed, regardless of their alignment with the field (Fig. 2A, RT = 80 min, 160 min).

Stumps cut in the median region of the meristem (*d* = 160 μm ± 10 μm) and exposed 30 min after excision also regenerated with significantly higher frequency compared to their mock, both with aligned (RR = 1.32, CI_RR_ = [1.19, 1.55]) and anti‐aligned (RR = 1.26, CI_RR_ = [1.17, 1.58]) fields (Fig. 2B, RT = 30 min). Remarkably, when we allowed roots to recover for a longer time from the median excision before imposing the field, we observed an increase of regeneration frequency in the aligned configuration (RR = 1.19, CI_RR_ = [1.08, 1.30]) but not in the anti‐aligned one (Fig. 2B, RT = 80 min). Similarly, but somehow with an opposite trend, after an even longer recovery time we observed an increase of regeneration frequency in the anti‐aligned configuration (RR = 1.25, CI_RR_ = [1.07, 1.40]) but not in the aligned one (Fig. 2B, RT = 160 min). These results suggest preferred polarities built within the unknown mechanism that determines the temporal competence to respond to the field.

Finally, none of the roots cut in the distal region of the meristem (*d* = 120 μm ± 10 μm) seemed to respond to the electric stimulation, regardless of the resting time RT or their alignment to the field (Fig. 2C). It should be noted that in this region the regeneration frequency in mock conditions was already high, making the detection of any potential increase quite hard.

### A weak electric field does not perturb tissue reorganization during regeneration

To observe at the cellular level the effects of the external electric field on the regeneration process, we imaged once a day individual regenerating roots through laser scanning confocal microscopy, following established methods (Sena et al. 2009). This generated temporal series of median optical sections, conveying information on the dynamics of the process for individual roots that did successfully regenerate, after 30‐min electric field exposure or mock conditions.

**Figure 3:**
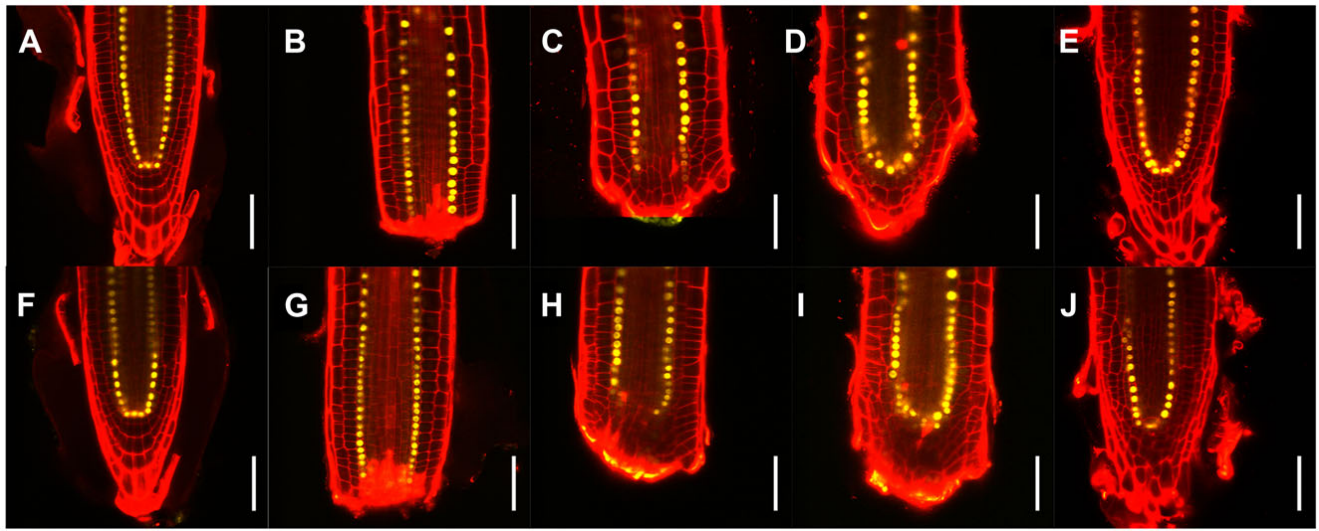
Effects of electric field on morphology and SCR expression pattern during root tip regeneration. Temporal series of longitudinal median optical sections of representative root meristems expressing *pSCR::H2B::YFP*. Mock treatment (A)−(E) and aligned 2.5 V/cm electric field exposure (F)−(J) are shown, for both uncut roots (A, F) and individual regenerating roots at 0 (B, G), 1 (C, H), 2 (D, I), and 4 (E, J) days after treatment. Yellow, YFP signal; red, propidium iodide counterstain. Scale bars: 50 μm.

We first asked if the overall morphology of tissue reorganization in the root meristem is perturbed by the electric field, in conditions that determined a significant increase of regeneration frequency: excision in the median position (160 μm ± 10 μm from the tip), RT = 30 min, aligned 2.5 V/cm electric field. The transcription factor SCARECROW (SCR) is a key regulator for radial pattern formation in *Arabidopsis* root, as well as in the establishment of the stem cell niche (Di Laurenzio et al. 1996; Sabatini et al. 2003). We used the nuclear‐localized transcriptional reporter *pSCR::H2B::YFP* (Heidstra et al. 2004) to observe the regeneration of its expression pattern with (Fig. 3F−J) and without (Fig. 3A−E) exposure to the electric field. No obvious differences were observed between the two treatments, both at the simple morphological level and in the re‐establishment of *SCR* expression pattern.

### A weak electric field inhibits cell divisions in the early steps of regeneration

In order to quantify potential effects of the electric field on cell proliferation in the regenerating tissue, we imaged roots expressing the mitotic cyclin reporter *CYCB1;1::GFP* (Colon‐Carmona et al. 1999; Reddy et al. 2004), under the same conditions described for *pSCR::H2B::YFP*. We focused on the early steps of regeneration, collecting *z*‐stacks of optical sections of roots either within 1 h or between 2 and 4 h after mock treatment or electric field exposure (Fig. S4). We calculated the mitotic index as the ratio between the number of cells expressing *CYCB1;1::GFP* in the whole *z*‐stack and the total number of cells considered (see Material and Methods).

**Figure 4:**
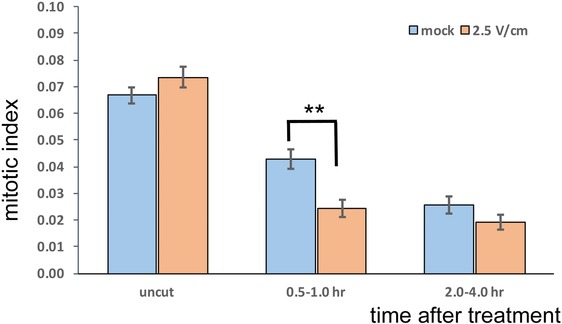
Effects of electric field on mitotic index during root tip regeneration. Number of cells expressing *CYCB1;1::GFP* divided by the total number of cells considered (mitotic index), in regenerating root meristems. Full three‐dimensional stacks were collected at each time‐point (not shown). Mock treatment and aligned 2.5 V/cm electric field exposure are presented color‐coded, for uncut roots and regenerating roots imaged between 0.5 and 1.0 h and between 2.0 and 4.0 h after treatment. Chi‐squared test was used to compare proportions; **high significance with *P* < 0.01; error bars, standard error of the proportion.

Surprisingly, our results indicate that within the first hour after treatment the aligned 2.5 V/cm electric field significantly inhibits cell divisions, compared to mock conditions, and that this effect becomes undetectable already after another hour (Fig. 4).

### A weak electric field perturbs auxin, but not cytokinin, distribution

The complex network of molecular regulators of cell divisions in the root meristem of *Arabidopsis* is not fully understood, but it is clear that a number of plant‐specific hormones play key roles in the controlled balance between cell division and differentiation (Pacifici et al. 2015; Fisher & Sozzani 2016). Auxin and cytokinin are possibly the best studied cross‐interacting plant hormones, shown to be involved in the establishment and maintenance of the root apical meristem (Ioio et al. 2007; Schaller et al. 2015). Interestingly, it has been previously suggested that the transport of auxin might be perturbed by weak electric fields (Morris 1980).

We investigated the possibility of perturbed auxin and cytokinin distributions in the root tip after exposure to the same weak electric field that produced increased regeneration frequency and reduced mitotic index.

**Figure 5:**
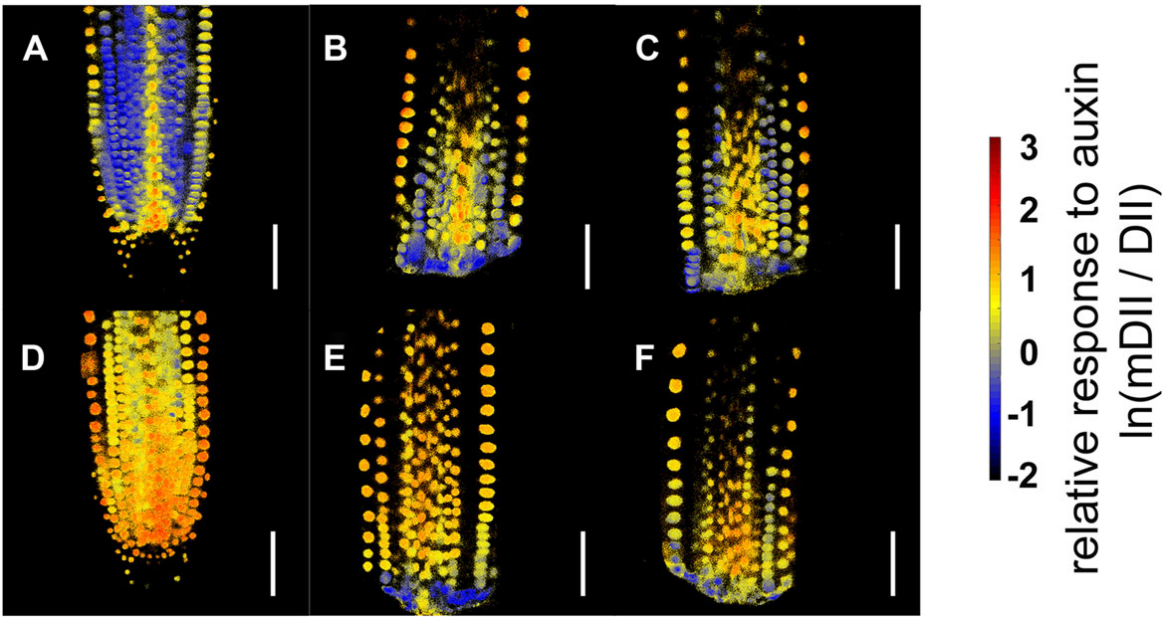
Effects of electric field on auxin distribution during root tip regeneration Longitudinal median optical sections of representative root meristems expressing *R2D2* reporter. Mock treatment (A)−(C) and aligned 2.5 V/cm electric field exposure (D)−(F) are shown, for both uncut roots (A, D) and regenerating roots imaged between 0.5 and 1.0 h (B, E) and between 2.0 and 4.0 h (C, F) after treatment. Scale bars: 50 μm.

The novel ratiometric fluorescence reporter *R2D2* (Liao et al. 2015) was used to quantify the local accumulation of auxin (Fig. S5), under the same conditions as described above for the *CYCB1;1::GFP* line: excision in the median position (160 μm ± 10 μm from the tip), RT = 30 min, aligned 2.5 V/cm electric field. Remarkably, uncut roots exposed to the field exhibited an increased accumulation of auxin in all tissues of the root meristem compared to mock conditions (Fig. 5A, D). This difference appears less dramatic but still detectable in regenerating stumps within the first hour after they have been cut and exposed to the field (Fig. 5B, E), but seems to disappear an hour later (Fig. 5C, F).

**Figure 6:**
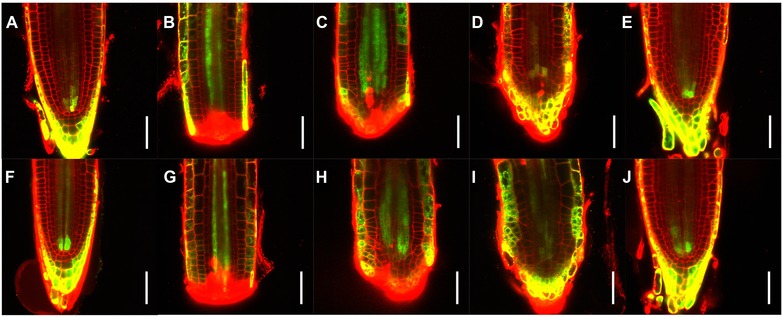
Effects of electric field on cytokinin distribution during root tip regeneration. Temporal series of longitudinal median optical sections of representative root meristems expressing *TCSn::GFP*. Mock treatment (A)−(E) and aligned 2.5 V/cm electric field exposure (F)−(J) are shown, for both uncut roots (A, F) and individual regenerating roots at 0 (B, G), 1 (C, H), 2 (D, I), and 4 (E, J) days after treatment. green, GFP signal; red, propidium iodide counterstain; yellow, overlapping GFP and PI signals. Scale bars: 50 μm.

To observe cellular activity in response to local accumulation of cytokinin, we used plants expressing the synthetic sensor *TCSn::GFP* (Zürcher et al. 2013) and generated temporal series of individual regenerating roots in the same conditions used for the auxin reporter *R2D2*. In uncut roots, the signal was strongest in columella cells, lateral root cap and vasculature initials, with no obvious difference between roots exposed to the field or in mock conditions (Fig. 6A, F). We also could not detect any difference between exposed and mock during regeneration (Fig. 6B−E versus 6G−J).

Together, these results indicate that auxin distribution in the root meristem is perturbed by the aligned 2.5 V/cm electric field, but that cytokinin distribution is not.

## Discussion

### External electric fields and tissue self‐organization

This work represents an original study on the manipulation of *in planta* organ regeneration through an external electric field, and more in general a step forward towards a better understanding of tissue self‐organization. More work is required to pinpoint the cellular mechanism acted upon by the electric field.

Early studies indicated that the developmental polarity of single cells can be modified with exposure to electric fields, as first shown in the egg cell of the brown alga *Focus serratus* (Lund 1923; Novák & Bentrup 1973; Peng & Jaffe 1976) and later observed in many other eukaryotic single cells (Lund 1925; Jaffe & Nuccitelli 1977). More recent work showed that imposed external electric fields are in some instances sufficient to change cellular orientation, expansion, proliferation, migration, and differentiation in *Dyctostelium discoideum*, fission yeast, and animal tissues (Chang & Minc 2014). These studies clearly indicate that electric fields are capable of perturbing cellular and developmental processes in a wide spectrum of tissues and organisms.

**Figure 7:**
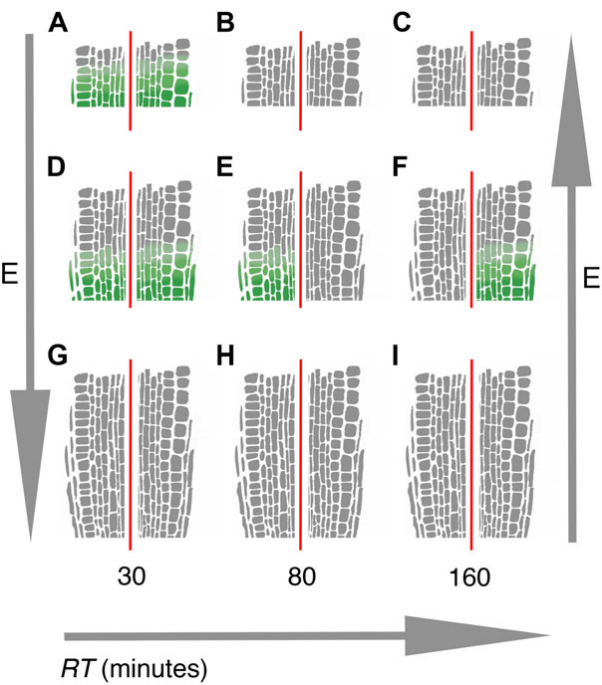
Summary of tissue competence to respond to the imposed electric field. Root schematics indicating in green high competence to respond to the imposed electric field (*E*); the half roots on the left/right of the red line depict the aligned/anti‐aligned configuration. Proximal (A−C), median (D−F), and distal (G−I) excisions are depicted, for resting times RT equal to 30 (A, D, G), 80 (B, E, H), and 160 (C, F, I) min.

Our results indicate that in *Arabidopsis* the regeneration process *in planta* of the root apical meristem can be perturbed by a brief exposure of the stump to a weak external electric field. Our estimates show that the probability of regeneration can increase up to more than two‐fold*_,_* under the appropriate conditions, as indicated by the 95% confidence intervals of the regeneration ratio, CI_RR_. Interestingly, the magnitude of this effect changes both in space (how far from the tip is the excision) and in time (how much time elapsed between the cut and the exposure), suggesting a tight regulation on spatial and temporal competence to respond to the applied electric field (summarized in Fig. 7). Our data indicate that the youngest and most distal region of the meristem is the least susceptible to the action of the imposed field (Fig. 7G−I), that the median region is the most likely to respond (Fig. 7D−F), and that the more mature and proximal region of the meristem remains susceptible for at least 30 min but no longer than 80 min after the excision (Fig. 7A−C).

It should be noted that cells in the proximal zone of the meristem are on average more mature and differentiated than the cells in the median zone. Also, in the early steps of regeneration cells in the stump are differentiating quite rapidly (Sena et al. 2009). An intriguing hypothesis is therefore that the cells in the median region and more than 80 min after the cut (Fig. 7E, F) are in a differentiation state comparable to those in the proximal region but only 30 min after the cut (Fig. 7A), and are therefore still susceptible to the external field. This suggests the existence of a narrow differentiation window (Fig. 7d,e,f,a), when the tissue is competent to respond to the electric field with an enhanced probability of regeneration.

A final crucial cue comes from the subtle difference we detect between aligned and anti‐aligned configurations (Fig. 7E, F, left versus right). These results seem to suggest that a polarized distribution of charges (ions, charged molecules, etc.) in the stump contributes to its regeneration, and that the external field might impose an optimal charge distribution in a time‐dependent manner (RT = 80 versus 160 min).

### The role of auxin and cell divisions

We have shown a simple correlation between increased auxin concentration, decreased cell division rate, and increased regeneration frequency after a brief exposure of excised root tips to a weak 2.5 V/cm electric field.

Interestingly, ectopic auxin localization has been shown to induce significant aberrations in the patterns of cell division in the *Arabidopsis* root meristem (Sabatini et al. 1999), so it is possible we detected some aspect of this. We currently do not have an explanation why reduced cell proliferation should correlate with increased regeneration frequency, so further investigation is needed.

As mentioned, it has already been proposed that auxin flow can be perturbed by external electric fields (Morris 1980), so we are currently pursuing potential molecular mechanisms. One intriguing possibility is that the field simply acts on charged proteins whose localization is functional to the transport of auxin, resulting in a perturbed distribution of auxin somehow more likely to initiate tissue regeneration. After all, the frequency of regeneration of *Arabidopsis* roots not exposed to an electric field is highest when the excision is performed in the distal zone of the meristem (this work and Sena et al. 2009), which is exactly where auxin concentration is usually at its maximum (Liao et al. 2015).

### Competence and robustness

Our results indicate that the increased regeneration frequency induced by the electric field correlates with an early reduction of cell proliferation and an early increase of auxin concentration, but without obvious changes at the tissue‐patterning level later during regeneration. This seems to suggest that the external field is sufficient to increase the tissue regeneration competence, possibly by perturbing auxin distribution, cell division rates, or both, but that once regeneration is initiated the process is robust to external perturbations.

We are currently investigating the molecular mechanisms involved in both of these developmental aspects of tissue regeneration in plants.

## Materials and Methods

### Plant material

The regeneration frequency experiments were performed with wild‐type *Arabidopsis thaliana* Columbia (Col‐0) ecotype. The reporter lines expressing *CYCB1;1::GFP* (Reddy et al. 2004), *pSCR::H2B:YFP* (Heidstra et al. 2004), and *TCSn::GFP* (Zürcher et al. 2013) are in wild‐type *Arabidopsis thaliana* Columbia (Col‐0) ecotype background. The reporter line expressing *RPS5A::mDII:ntdTomato‐RPS5A::DII:n3Venus*, also known as *R2D2* reporter (Liao et al. 2015), is in wild‐type *Arabidopsis thaliana* Columbia Utrecht (Col‐utr) ecotype background.

Seeds were imbibed in water and synchronized for 2 days at 4°C and in darkness. Seeds were surface‐sterilized in 50% Haychlor bleach (sodium hypochlorite) (Brenntag, UK) and 0.0005% Triton X‐100 (Sigma‐Aldrich, UK) for 3 min and rinsed six times in sterile water. Sterile seeds were then transferred under sterile conditions to standard 0.8% agar solid medium (1× Murashige and Skoog Basal medium (Sigma‐Aldrich, UK), 0.5% sucrose (Sigma‐Aldrich, UK), 0.05% 2‐morpholin‐4‐ylethanesulfonic acid [MES hydrate (Sigma‐Aldrich, UK)], adjusted to pH 5.7 with KOH (Sigma‐Aldrich, UK)) and germinated in a vertical position in a plant growth chamber with 120 μmol/m^2^/s light intensity on a 16‐h light/8‐h dark cycle and constant 23°C.

### Root meristem excision

All root tip excisions were performed on primary roots of 3‐day post‐germination seedlings, in accordance with an established protocol (Sena et al. 2009). Seedlings were transferred under sterile conditions onto 5.0% agar solid medium (1× Murashige and Skoog Basal medium, 0.5% sucrose, 0.05% MES hydrate, adjusted to pH 5.7 with KOH) and root tips were manually dissected with 100 Sterican 27G needles (B Braun) under a dissecting stereo‐microscope (Nikon SMZ1000 at 180× magnification). The excisions were performed at 120 μm, 160 μm, and 200 μm from the tip, with an estimated error of ± 10 μm.

### Electric field exposure

Before exposure, plantlets were moved onto an agar slab modified with a pillow‐like support spread on top of it (Fig. S1), both made of 0.8% agar solid medium (1× Murashige and Skoog Basal medium, 0.5% sucrose, 0.05% MES hydrate, adjusted to pH 5.7 with KOH). The whole slab was positioned horizontally in a Vari‐Gel (Denville Scientific) electrophoresis tank, with the roots parallel to the direction of the electric field. The tank was slowly filled with 550 mL of sterile liquid MS medium (1× Murashige and Skoog Basal medium, 0.5% sucrose, 0.05% MES hydrate, adjusted to pH 5.7 with KOH), with the roots completely submerged and the shoots resting on the pillow‐like support to keep the leaves above the liquid at all times (Fig. S1). In order to maintain a uniform distribution of ions in the medium and constant temperature, the liquid medium was continuously circulated with a small peristaltic pump (Uno International, UK) at 40 mL/min from the side of the positive electrode (Fig. S1). Part of the silicone tubing used to circulate the medium was immersed in an LTD6/20 chiller (Grant Instruments, UK) to maintain a constant 23°C in the tanks during the experiment (Fig. S1). The electric field was kept constant for 30 min. After the electrical stimulation, seedlings were transferred onto fresh standard 0.8% agar solid medium in square plastic plates.

### Regeneration frequency assay

The square plastic plates from the previous step were placed vertically in the same growth chamber used for seed germination but rotated 90^o^ so that the roots were now oriented perpendicularly to the gravity vector. Seedlings were allowed to regenerate for 5 days before being scored for gravity stimulation in accordance with previous methods (Sena et al. 2009). Scoring for positive regeneration was performed under blind conditions, where the tester was not aware whether the roots were mock or exposed. Re‐established positive gravitropism was used as proxy for root tip regeneration (Sena et al. 2009) (see above), confirmed by morphological examination under a dissecting microscope (Nikon SMZ1000 at 22× magnification). The frequency of regeneration was estimated as the fraction of the cut roots that successfully regenerated at 5 days post‐cut.

### Confocal imaging

Individual roots were imaged during regeneration at the time steps indicated above, following established methods (Sena et al. 2009). Each root shown in the figures is representative of at least three observed roots.

After exposure to the electric field, roots expressing *p35S::CYCB:GFP*, *pSCR::H2B:YFP*, and *TCSn::GFP* were counterstained in red with 10 μg/mL propidium iodide (Sigma) for 3 min and then briefly washed with sterile water. The counterstain is excluded from healthy cell membranes and shows cell shapes. Roots expressing *R2D2* were not counterstained. The roots were mounted with sterile water and imaged using a Leica SP5 laser scanning confocal microscope with a 63× objective. The *R2D2* reporter was excited with 514 nm and 543 nm wavelengths in sequence, following the published protocol (Liao et al. 2015), and its emission was collected between 524 and 570 nm for yellow fluorescence and between 580 and 630 nm for red fluorescence using HyD (Leica) detectors. The *pSCR::H2B:YFP* reporter was excited with 514 nm wavelength and emission was collected between 524 and 570 nm using HyD (Leica) detectors. The *TCSn::GFP* reporter was excited with 488 nm wavelength and emission was collected between 495 and 550 nm using HyD (Leica) detectors. The *p35S::CYCB:GFP* reporter was imaged using a Leica SP5 confocal microscope with resonant scanner, using an excitation of 488 nm wavelength and collecting emission between 495 and 550 nm. *Z*‐stack images were collected with 3 μm steps. Propidium iodide counterstain was excited with either 488 nm or 514 nm wavelength and emission was collected between 580 and 630 nm.

Between observations, individual plants were transferred from the microscope slide onto 0.8% agar solid MS medium and grown under standard conditions (16‐h light/8‐h dark, 120 μmol/m^2^/s light intensity, 23°C).

### Ratiometric analysis of *R2D2*

The ratio of the two channels for each *R2D2* image was calculated in MATLAB (The MathWorks Inc.). In each channel, the background mean and standard deviation were calculated from an 80 × 80 pixel area. The background mean was subtracted from the full image, and all pixels with intensity lower than 25 times the background's standard deviation were set to the minimum. Finally, the natural logarithm of the ratio between the *mDII:ntdTomato* and *DII:n3Venus* was rendered in a new image, color‐coded to show the full range of values.

### Mitotic index

*Z*‐stack images of each primary root expressing *p35S::CYCB::GFP* were analyzed with the help of Fiji (Schindelin et al. 2012) to count the number of GFP+ cells in the field of view, considering only epidermis, cortex, and endodermis tissues. The total number of cells considered in the three‐dimensional stack was estimated by multiplying the number of epidermal, cortical, and endodermal cells seen in each median section by the stereotypical number of cells in the circumference of each of the three tissues (Dolan et al. 1993): 16 cells for epidermis, eight for cortex, and eight for endodermis. A mitotic index for each root was then calculated as the ratio between the observed number of GFP+ cells and the total number of cells estimated.

### Statistics

#### Regeneration frequencies

All the *n* replicates for each condition (at least three biological replicates) were organized in an *n* × 2 contingency table with categories *regenerated* and *not‐regenerated*. A chi‐squared statistical test was performed within each condition, to test the null hypothesis of no association between replicates and categories (i.e., replicates are samples drawn from the same population), as expected (Everitt 1992). Whenever the above null hypothesis was rejected with a conservative *P* < 0.05, a standard analysis of the residuals was performed to identify and purge any outlier replicate one at a time (adjusted residual bigger than the critical value 1.96 or smaller than −1.96, for α = 0.05) (Everitt 1992). For each condition, all remaining replicates were pooled together to increase the sample size while at the same time maintaining the biological variability. The frequency of regeneration was estimated as the proportion *f* of cut roots that regenerated in the sample considered, that is, the number of regenerated roots divided by the total number *N* of cut roots. The error of such estimate was quantified with the standard error of a proportion, defined as [*f*(1 – *f*)/*N*]^1/2^ (Glantz 2005). All our samples showed very similar standard errors of a proportion, indicating analogous estimates of the variance of underlying populations.

In order to compare two independent regeneration frequencies a 2 × 2 contingency table was built and the chi‐squared test was used to test the association between condition (e.g., mock versus exposed) and category (regenerated versus not‐regenerated) (Everitt 1992). The null hypothesis (no association between condition and category) was rejected for *P* < 0.01, and a statistically significant difference between the regeneration frequencies in the two conditions was declared. In the case of multiple tests, for example when a single mock sample was used twice to be compared to aligned and anti‐aligned conditions, the critical value for high significance was corrected from *P* = 0.01 to *P* = 0.01/2 = 0.005, according to the standard Bonferroni inequality (Glantz 2005). In order to have a chi‐squared test with significance level (false positive probability) <0.05, power (true positive probability) >0.8 and effect size *w* = 0.15 (to detect small differences), the minimum number of roots to consider in a single 2 × 2 contingency table was estimated as *N* = 348 (*pwr.chisq.test* function in the *pwr* package of the R statistics computing environment) (RDevelopmentCoreTeam 2011) (Table S1).

The regeneration ratio is completely equivalent to the risk ratio or relative risk commonly used in epidemiology studies and was calculated as the ratio between the regeneration frequency in exposed and mock conditions, together with its 95% confidence interval (Glantz 2005) (*riskratio* function in the *epitools* package of the R statistics computing environment) (RDevelopmentCoreTeam 2011).

#### Mitotic index

The mitotic index was calculated for each root expressing *CYCB1;1::GFP* as the ratio between the number of GFP+ cells and the total number of cells considered in epidermis, cortex, and endodermis tissues only. Analogously to what was done with the regeneration frequencies, all biological replicates for each condition were organized in an *n* × 2 contingency table with categories GFP+ and GFP−, and the same standard analysis of the residuals described above was performed to identify and purge any outlier replicate one at a time (Everitt 1992). All the comparisons between two conditions were done with a chi‐squared test in the 2 × 2 contingency table, and a significant difference was accepted for *P* < 0.01.

## Competing interests

The authors declare no competing financial interests.

## Supporting information

Additional Supporting Information may be found in the online version of this article at the publisher's website:

Additional Supporting Information may be found in the online version of this article at the publisher's website:
**Table S1**. Total number of roots cut in each condition
Each row contains the total number of cut roots considered for proximal, median and distal regions, respectively. Columns are grouped in *RT* = 30, 80 and 160 minutes, and represent the three experimental conditions: mock, aligned and antialigned. Top, E = 2.5 V/cm; bottom, E = 5.0 V/cm.

**Figure S1**. Schematic and picture of the setup
**(a,b)** Cartoons showing the arrangement of plantlets during exposure to the electric field, in the aligned configuration. **(a)** lateral view; **(b)** top view. **(c)** Picture of actual electrophoresis tank and plantlets positioned on the gel. **Sm,** solid medium; **Lm,** liquid medium; **Su,** pillow‐like support; **Lv,** leaves; **Rt,** root.

**Figure S2**. Root tip regeneration frequencies in absence of electric field (mock)
Number of regenerated roots divided by the total number (reported in Table S1) of root tips cut at the distal, median and proximal positions. Resting times *RT* = 30, 80 and 160 minutes are presented colour‐coded. Error bars, standard error of the proportion.

**Figure S3**. Effects of strong electric field on the frequency of root tip regeneration
Number of regenerated roots divided by the total number (reported in Table S1) of root tips cut at the median positions and exposed to 5.0 V/cm, for resting time *RT* = 80’. Mock, aligned and antialigned configurations are presented colour‐coded. Chi‐square tests to compare proportions; ** high significance with *P* < 0.005 Bonferroni‐corrected; error bars, standard error of the proportion.

**Figure S4**. Effects of electric field on cell divisions during root tip regeneration
Longitudinal median optical sections of representative root meristems expressing *CYCB1;1::GFP*. Mock treatment **(a‐c)** and aligned 2.5 V/cm electric field exposure **(d‐f)** are shown, for both uncut roots **(a, d)** and regenerating roots imaged between 0.5 and 1.0 hours **(b,e)** and between 2.0 and 4.0 hours **(c,f)** after treatment. Green, GFP signal; red, Propidium Iodide counterstain. Scale bars, 50μm.

**Figure S5**. Ratiometric analysis of *R2D2* reporter
The two components of the *R2D2* reporter are imaged and analysed in an uncut root **(a‐d),** and a single root expressing *R2D2* is shown during regeneration **(e‐h)**. **(a)** *RPS5A::mDII::ntdTomato* (*mDII*, control for expression), **(b)** *RPS5A::DII::n3xVenus* (*DII*, inverse proportional to auxin concentration), **(c)** overlap of the two previous signals, **(d)** ln(*mDII / DII*) with colour‐coded scale. The regenerating root is shown at 0 **(e),** 1 **(f),** 2 **(g)** and 4 **(h)** days after excision. Scale bars, 50μm.
