# Supplementary material for: Externally imposed electric field enhances plant root tip regeneration
Source: Regeneration (Oxf). 2016 Aug 20;3(3):156–67. doi: 10.1002/reg2.59 (PMC5011479; doi:10.1002/reg2.59)
Supplement: Supplementary file 1 — Additional Supporting Information may be found in the online version of this article at the publisher's website: Table S1. Total number of roots cut in each condition Each row contains the total number of cut roots considered for proximal, median and distal regions, respectively. Columns are grouped in RT = 30, 80 and 160 minutes, and represent the three experimental conditions: mock, aligned and antialigned. Top, E = 2.5 V/cm; bottom, E = 5.0 V/cm. [file REG2-3-156-s001.docx]

|  | resting time (*RT*) | | | | | | | | |
| --- | --- | --- | --- | --- | --- | --- | --- | --- | --- |
| ***2.5 V/cm*** | 30 min | | | 80 min | | | 160 min | | |
|  | mock | aligned | antialigned | mock | aligned | antialigned | mock | aligned | antialigned |
| proximal | 237 | 163 | 255 | 430 | 208 | 181 | 367 | 160 | 194 |
| median | 307 | 195 | 269 | 663 | 420 | 215 | 346 | 202 | 234 |
| distal | 175 | 181 | 193 | 285 | 251 | 239 | 321 | 233 | 209 |
|  |  |  |  |  |  |  |  |  |  |
|  | resting time (RT) | | |  |  |  |  |  |  |
| ***5.0 V/cm*** | 80 min | | |  |  |  |  |  |  |
|  | mock | aligned | antialigned |  |  |  |  |  |  |
| median | 613 | 44 | 50 |  |  |  |  |  |  |

**Table S1**

Total number of roots cut in each condition
