# Supplementary figures and images for: Externally imposed electric field enhances plant root tip regeneration
Source: Regeneration (Oxf). 2016 Aug 20;3(3):156–67. doi: 10.1002/reg2.59 (PMC5011479; doi:10.1002/reg2.59)

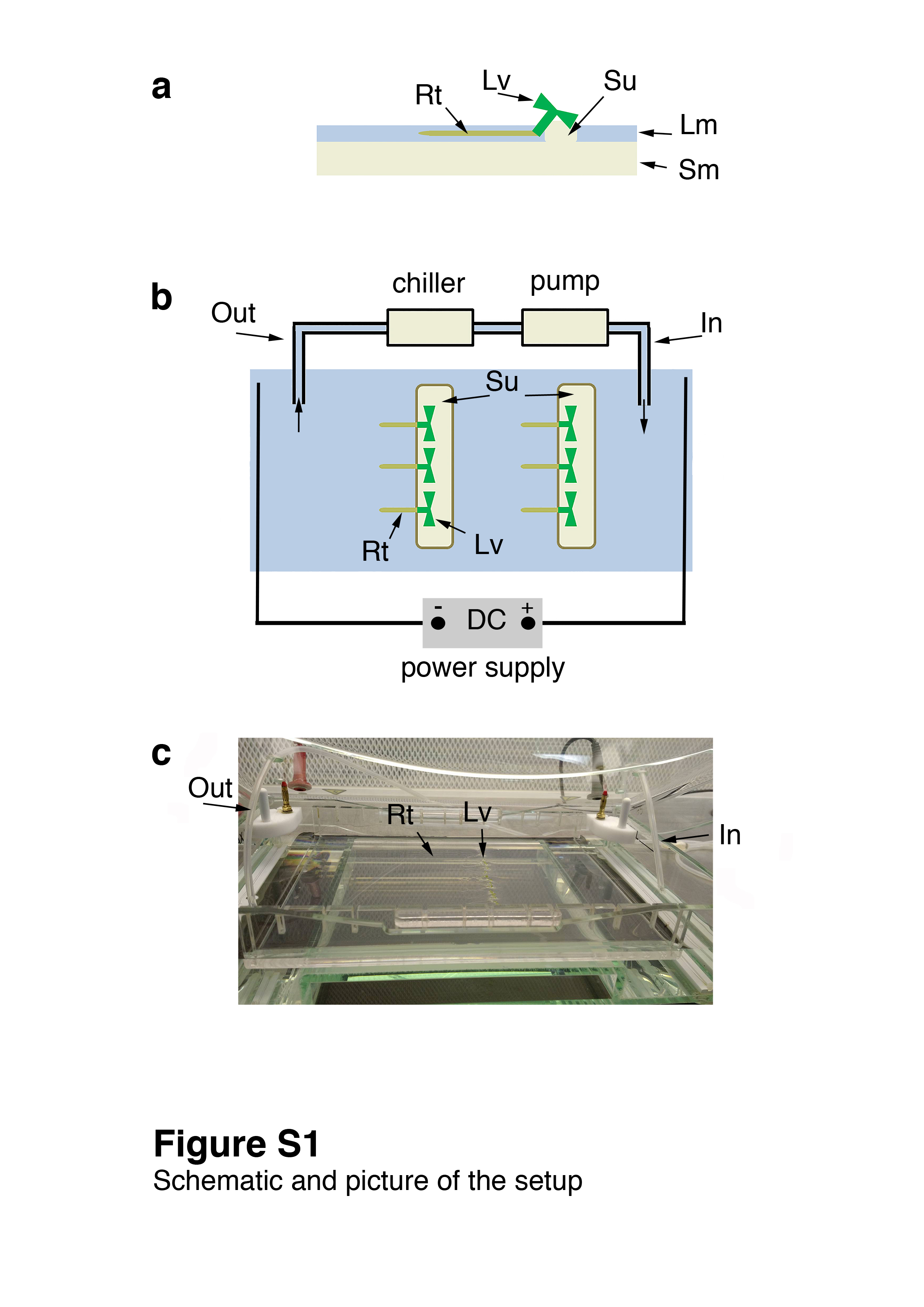

Supplement: Supplementary file 2 — Figure S1. Schematic and picture of the setup (a,b) Cartoons showing the arrangement of plantlets during exposure to the electric field, in the aligned configuration. (a) lateral view; (b) top view. (c) Picture of actual electrophoresis tank and plantlets positioned on the gel. Sm, solid medium; Lm, liquid medium; Su, pillow‐like support; Lv, leaves; Rt, root. [file REG2-3-156-s002.tiff]

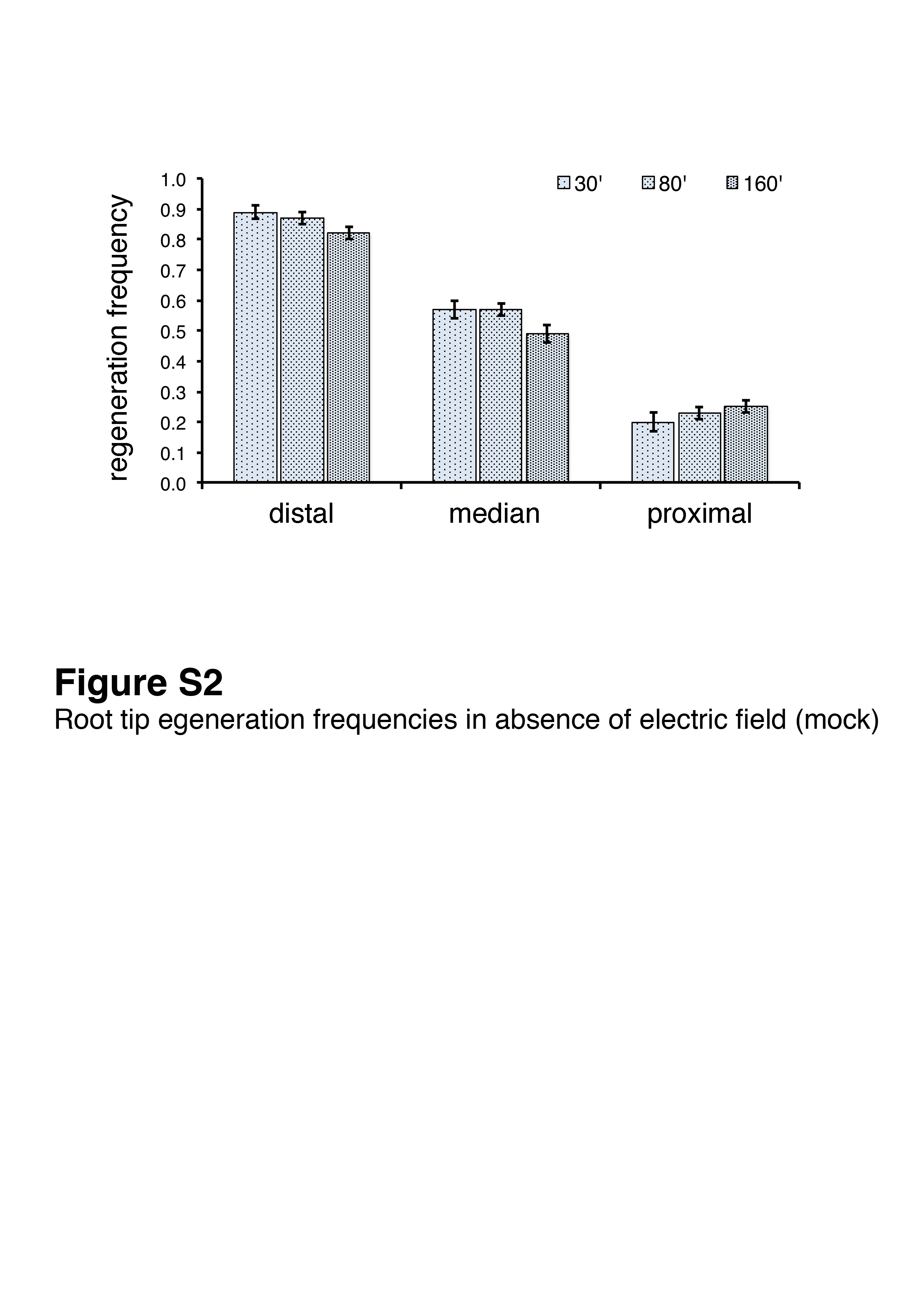

Supplement: Supplementary file 3 — Figure S2. Root tip regeneration frequencies in absence of electric field (mock) Number of regenerated roots divided by the total number (reported in Table S1) of root tips cut at the distal, median and proximal positions. Resting times RT = 30, 80 and 160 minutes are presented colour‐coded. Error bars, standard error of the proportion. [file REG2-3-156-s003.tiff]

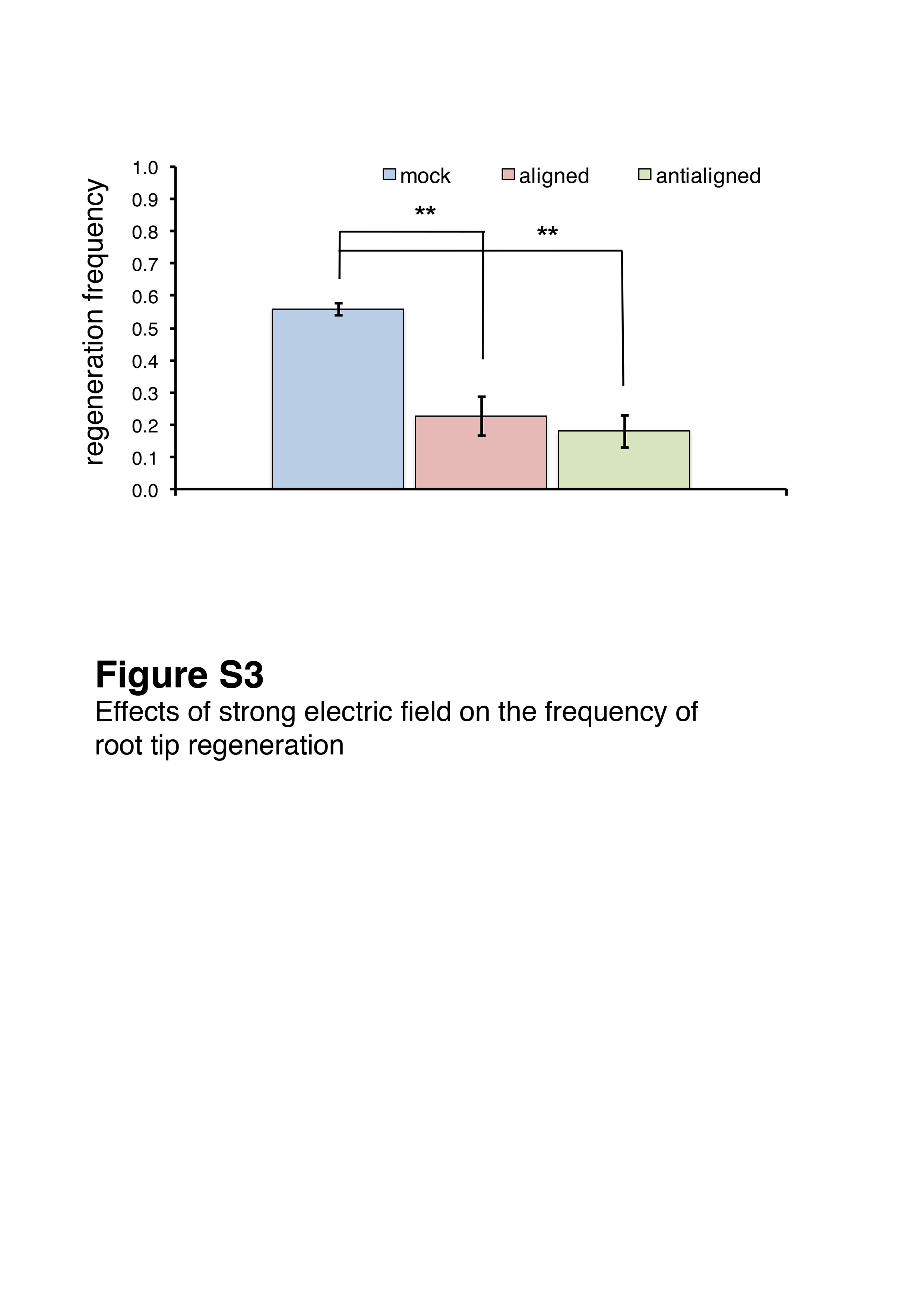

Supplement: Supplementary file 4 — Figure S3. Effects of strong electric field on the frequency of root tip regeneration Number of regenerated roots divided by the total number (reported in Table S1) of root tips cut at the median positions and exposed to 5.0 V/cm, for resting time RT = 80’. Mock, aligned and antialigned configurations are presented colour‐coded. Chi‐square tests to compare proportions; ** high significance with P < 0.005 Bonferroni‐corrected; error bars, standard error of the proportion. [file REG2-3-156-s004.tiff]

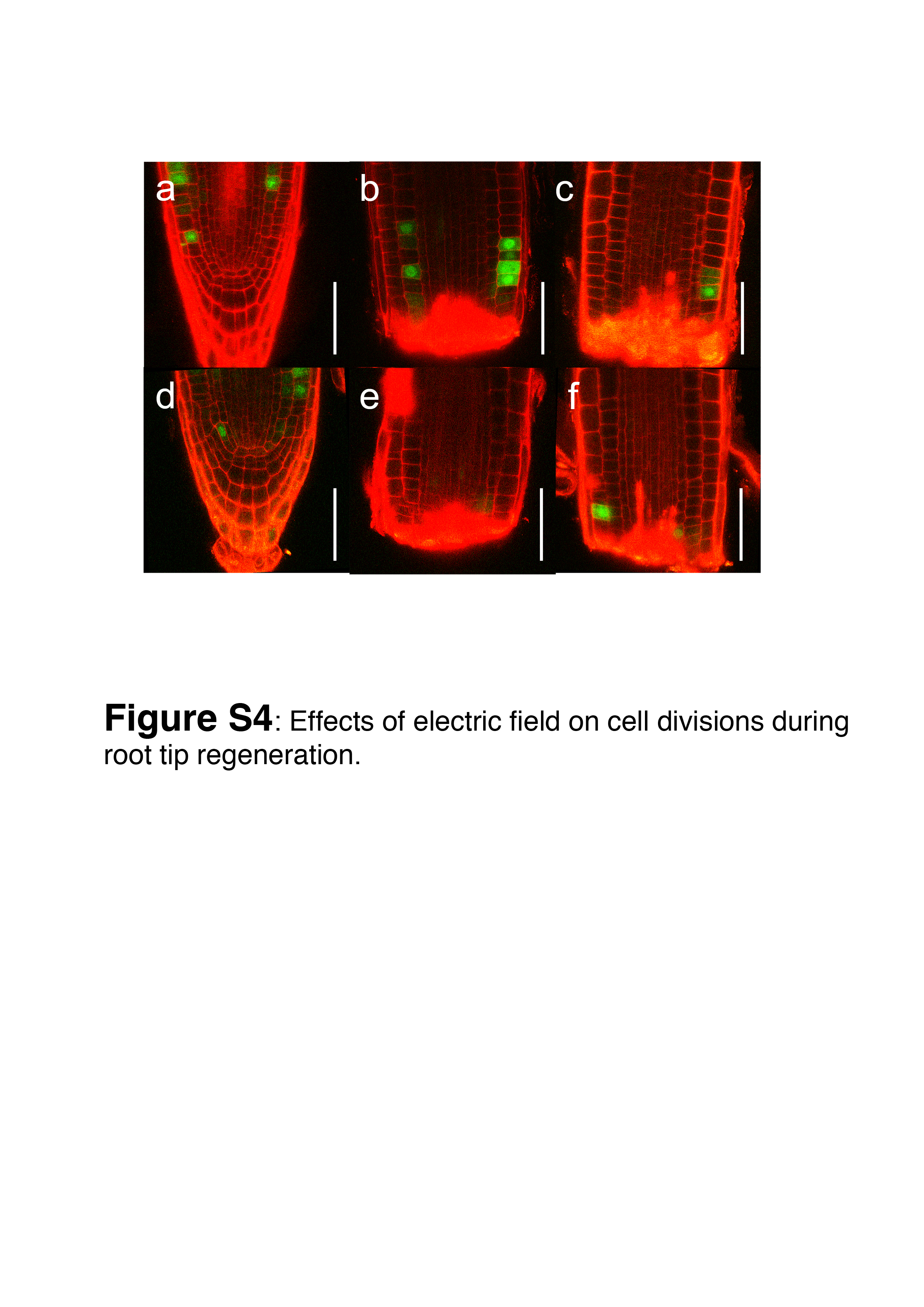

Supplement: Supplementary file 5 — Figure S4. Effects of electric field on cell divisions during root tip regeneration Longitudinal median optical sections of representative root meristems expressing CYCB1;1::GFP. Mock treatment (a‐c) and aligned 2.5 V/cm electric field exposure (d‐f) are shown, for both uncut roots (a, d) and regenerating roots imaged between 0.5 and 1.0 hours (b,e) and between 2.0 and 4.0 hours (c,f) after treatment. Green, GFP signal; red, Propidium Iodide counterstain. Scale bars, 50μm. [file REG2-3-156-s005.tiff]

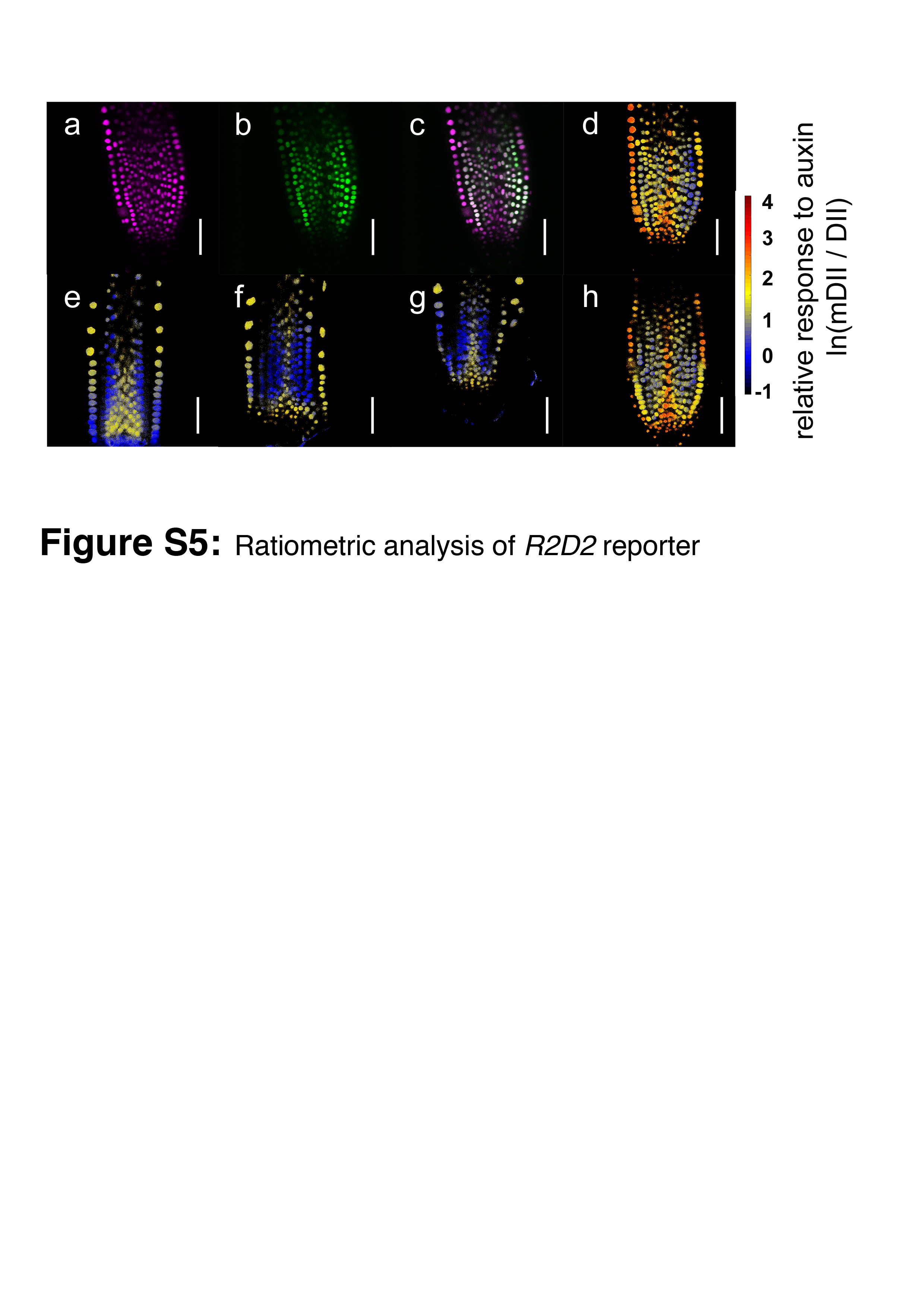

Supplement: Supplementary file 6 — Figure S5. Ratiometric analysis of R2D2 reporter The two components of the R2D2 reporter are imaged and analysed in an uncut root (a‐d), and a single root expressing R2D2 is shown during regeneration (e‐h). (a) RPS5A::mDII::ntdTomato (mDII, control for expression), (b) RPS5A::DII::n3xVenus (DII, inverse proportional to auxin concentration), (c) overlap of the two previous signals, (d) ln(mDII / DII) with colour‐coded scale. The regenerating root is shown at 0 (e), 1 (f), 2 (g) and 4 (h) days after excision. Scale bars, 50μm. [file REG2-3-156-s006.tiff]
